# Ultrasound–Histopathological Presentation of Thyroid and Ovary Lesions in Adolescent Patients with DICER1 Syndrome: Case Reports and Literature Overview

**DOI:** 10.3390/children11040403

**Published:** 2024-03-28

**Authors:** Dominika Januś, Monika Kujdowicz, Konrad Kaleta, Kamil Możdżeń, Jan Radliński, Anna Taczanowska-Niemczuk, Aleksandra Kiszka-Wiłkojć, Marcin Maślanka, Wojciech Górecki, Jerzy B. Starzyk

**Affiliations:** 1 Department of Pediatric and Adolescent Endocrinology, Jagiellonian University Medical College, 31-008 Krakow, Poland; jerzy.starzyk@uj.edu.pl; 2 Department of Pediatric and Adolescent Endocrinology, University Children’s Hospital in Krakow, 30-663 Krakow, Poland; 3 Department of Pathomorphology, Jagiellonian University Medical College, 31-008 Krakow, Poland; monika.kujdowicz@uj.edu.pl; 4 Department of Pathology, University Children’s Hospital in Krakow, 30-663 Krakow, Poland; 5 Student Scientific Group of Pediatric Endocrinology, Department of Pediatric and Adolescent Endocrinology, Jagiellonian University Medical College, 31-008 Krakow, Poland; konrad.kaleta@student.uj.edu.pl (K.K.); kamil.mozdzen@student.uj.edu.pl (K.M.); jan.radlinski@student.uj.edu.pl (J.R.); 6 Department of Pediatric Surgery, Jagiellonian University Medical College, 31-008 Krakow, Poland; anna.taczanowska-niemczuk@uj.edu.pl (A.T.-N.); aleksandra.kiszka-wilkojc@uj.edu.pl (A.K.-W.); marcin.maslanka@uj.edu.pl (M.M.); wojciech.gorecki@uj.edu.pl (W.G.); 7 Department of Pediatric Surgery, University Children’s Hospital in Krakow, 30-663 Krakow, Poland

**Keywords:** multinodular goiter, secondary amenorrhea, Sertoli–Leydig cell tumor, DICER1 syndrome, euthyroid goiter

## Abstract

Background: DICER1, a cancer predisposition syndrome (CPS), seems to escape timely diagnosis in pediatric patients. Case report 1: A 16-year-old female patient was referred to the endocrinology ward due to a large goiter. Her medical history indicated normal sexual maturation, with menarche occurring at 13.5 years. Over the past 2.5 years, she had developed pronounced androgenic symptoms, including a deepened male voice; facial, back, and neckline acne; hirsutism; and menstrual irregularities leading to secondary amenorrhea. A thyroid ultrasound identified a multinodular goiter (MNG) with cystic–solid lesions containing calcifications. An abdominal ultrasound identified a 5.7 × 6.9 cm solid mass in the right adnexal region, displacing the uterus to the left. Histopathological examination confirmed a Sertoli–Leydig cell tumor. The patient was subjected to a total thyroidectomy. Histopathology revealed benign follicular cell-derived neoplasms. Thyroid follicular nodular disease (TFND) was diagnosed bilaterally. DNA analysis using NGS, confirmed via the Sanger method, revealed a pathogenic heterozygotic variant c.2953C>T [p.Gln985*] in exon 18 of the *DICER1* gene. Case report 2: A 12-year-old male patient was admitted to the pediatric surgery unit due to a 33 mL goiter. A month prior to his admission, the patient discovered a palpable nodule in his neck, accompanied by hoarseness. An ultrasound revealed MNG. Molecular analysis revealed a pathogenic heterozygotic variant c.2782C>T [p.Gln928*] in exon 17 of the *DICER1* gene. Subsequently, a total thyroidectomy was performed, and histopathological examination revealed TFND bilaterally. Conclusions: Recent advances in genetic evaluation and in histological approaches indicate that MNG/TFND, although rare in the pediatric population, when accompanied by characteristic ultrasound and histopathological features, and by additional features such as androgenization, may warrant assessment also of the *DICER1* gene within CPS molecular panel screening.

## 1. Introduction

### DICER1 Syndrome

DICER1 syndrome is a cancer predisposition syndrome (CPS) caused by a heterozygous germline loss-of-function mutation in the *DICER1* gene (localized on 14q32.13) [1]. The enzyme coded by this gene, dicer (multi-domain endoribonuclease), identified by Bernstein et al. in 2001, modifies protein-coding genes by influencing the biogenesis of miRNAs [2]. *DICER1* is essential for embryogenesis and the development of many organs and is important in the oncogenesis of benign and malignant tumors [3].

In DICER1 syndrome, most tumors arise in patients with one inherited and one additional acquired somatic missense *DICER1* alteration [4,5,6,7,8,9,10,11,12]. The majority of alterations are inherited, with 10–20% being detected de novo [11]. The syndrome demonstrates an autosomal dominant inheritance pattern with reduced penetrance, subsequently decreasing the frequency of familial cases [9,10].

Mirshahi et al., using a genome-first approach, estimated that the prevalence of germline *DICER1* loss-of-function variants ranged from 1 in 3700 to 1 in 4600 people in 2021 [13]. This is more common than the frequency previously assumed to be around 1:10600 in 2017 [14].

Stewart et al. reported that the risk of developing a neoplasm is ~5% < 10 years of age and ~30% < 60 years of age in carriers of the pathogenic *DICER1* variant. This incidence is larger than in the general population [9,10].

DICER1 syndrome includes dysplastic and neoplastic lesions (with the tendency to develop sarcomas) [10]. It is usually associated with pleuropulmonary blastoma (PPB), a malignant tumor of the lungs in infants and children < 6 years of age, as well as cystic nephroma, ovarian Sertoli–Leydig cell tumors (SLCTs), and multinodular goiters (MNGs) [10].

**Figure 1:**
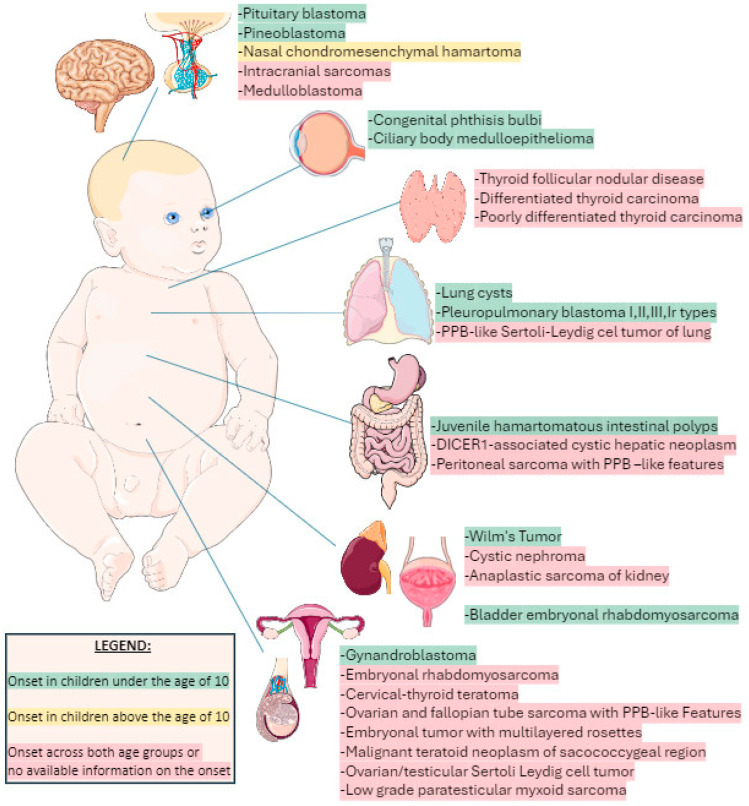
Age-dependent clinical manifestation of DICER1 syndrome. The colors represent the age of onset for various conditions, as detailed in the accompanying legend. Parts of the figure were drawn using pictures from Servier Medical Art. Servier Medical Art by Servier is licensed under a Creative Commons Attribution 3.0 Unported License (https://creativecommons.org/licenses/by/4.0/, accessed on 12 February 2024).

The clinical spectrum of this syndrome is quite broad, age-dependent, and likely still evolving. It involves many sites in the body and also includes such non-nodular features as macrocephaly, overgrowth, and eye and genitourinary problems, among others [1,2,3,4,5,6,7,8,9,10,15]. We present age-dependent comorbidities in Figure 1 [1,2,3,4,5,6,7,8,9,10,11,16].

The latest advancements in molecular studies on *DICER1* variants in relation to thyroid nodular disorders in childhood and adulthood indicate that thyroid disorders are the dominant manifestation of DICER1 syndrome [11,17,18].

As presented by Sauer et al., *DICER1* is important for normal thyroid development [18]. According to the 2022 World Health Organization Thyroid Tumor Classification, follicular cell-derived neoplasms are divided into benign (e.g., thyroid follicular nodular disease [TFND], follicular thyroid adenoma, follicular thyroid adenoma with papillary architecture), low-risk and malignant neoplasms, including differentiated thyroid carcinomas (DTCs), and poorly differentiated thyroid carcinomas (PDTCs) [19]. TFND and DTCs in infancy may raise the concern of possible germline, and PDTCs and thyroblastoma of possible somatic *DICER1* alteration [11,18].

The MNG, histopathologically assessed as TFND, is at present the most common manifestation of *DICER1* alterations [11,17,18]. The discovery of MNG/TFND in a pediatric patient especially may direct a clinician to evaluate the patient on a molecular level [11,17,18].

Khan et al. reported a higher MNG prevalence and penetrance (10–20%) among pathogenic *DICER1* variant carriers, with female predominance [20,21]. These pathogenic *DICER1* variant carriers also exhibit a 16- to 24-fold increase in thyroid carcinoma risk due to benign thyroid nodules that accumulate supplementary genetic abnormalities over time and eventually develop into malignant growths [20].

Pathogenic *DICER1* variant carriers are at increased risk of developing SLCTs and gynandroblastomas (tumors associated with androgen excess) from childhood to adulthood [22,23].

This study presents two case reports of adolescent patients from one center in southern Poland carrying pathogenic *DICER1* variants. In this article, we present specific clinical findings, as well as unique ultrasound and histopathological features of the tumors. Our goal is to raise awareness about this syndrome and underline the importance of not only a point-of-care ultrasonography in the pediatric office, as a tool that can potentially shorten the diagnostic process, but also of the significant role of the pathologist in this process.

## 2. Methods

The patients’ levels of TSH, fT3, fT4, LH, FSH, and testosterone were assessed via chemiluminescence (Advia Centaur XPT; Siemens, Berlin, Germany); estradiol, 17-OHP, thyroperoxidase (TPOAb), thyroglobulin (TgAb), and TSH receptor (TRAb) antibody were assessed via radioimmunoassay (BRAHMS, ThermoFisher, Scientific, Waltham, MA, USA). Ultrasound (US) assessment was performed by D.J., A.T.N. and A.K.W., certified in pediatric US (Philips IU22, Eindhoven, The Netherlands, and Samsung HS40, Samsung Healthcare, Suwon, Republic of Korea, ultrasound equipment, linear probes), and based on EU-TIRADS 2017 and PL 2022 scales [24,25]. For fine-needle aspiration biopsies, the Bethesda scale was used [26]. Total thyroidectomies with central lymph node histopathological verification and right ovariectomy were performed in the Departments of Pediatric Surgery and Pathology. As a control group for the comparison of ultrasound–histopathological correlations, we presented scans from two adolescent patients diagnosed with papillary thyroid carcinoma (*DICER1*-negative).

Molecular analysis of the blood samples was performed in a commercial molecular laboratory in Poland (with new-generation sequencing, NGS, and Sanger methods).

This study was approved by the relevant institutional review board (The Bioethics Committee of the Jagiellonian University; opinion number: 1072.6120.288.2021). Written informed consent was obtained from all participants and/or their parents. Written informed consent was obtained from the individuals, and the minors’ legal guardians, for the publication of any potentially identifiable images or data included in this article.

## 3. Case Report 1

A 16-year-old female patient was referred to the outpatient Pediatric and Adolescent Endocrinology Department for evaluation of her thyroid function due to a large goiter. The patient’s perinatal history was unremarkable (APGAR score of 10 points, birth weight of 3400 g), with normal developmental milestones. Her general health status was good, with no significant diseases reported to date. Laboratory results showed no abnormalities. However, during conversation with this patient, it was noticeable that her voice was deepened, resembling a male voice. Her medical history indicated normal sexual maturation, with menarche occurring at 13.5 years. Over the past 2.5 years, she had exhibited pronounced androgenic symptoms of defeminization, including decreased volume of the breasts (evidenced by her need to buy a smaller bra), balding, an enlarged clitoris, progressive deepening of her voice, facial, back, and neckline acne, a hirsutism score of 18 points according to the Ferriman–Gallwey scale (the Polish norm being < 8 points), and menstrual irregularities leading to secondary amenorrhea during the last 12 months. Additionally, she reported consistent genital spotting for the past two months but was not referred to a gynecologist. The patient had consulted with an otorhinolaryngologist twice over the past 1.5 years regarding her deepening voice, and since examination did not reveal any cause, the doctor ordered a testosterone assessment. The hormonal analysis showed elevated testosterone levels on two occasions (6.1 ng/mL and 4.17 ng/mL, with the norm for her demographic being less than 0.48 ng/mL). As the latter measurement was lower than the former, the patient did not receive any further referrals. Finally, because of the gradual enlargement of her thyroid, the large goiter started to become visible, and her family doctor referred the patient to a pediatric endocrinologist. This patient did not receive a referral to a gynecologist despite the presence of secondary amenorrhea. The evaluation in the outpatient Pediatric and Adolescent Endocrinology Department revealed that the patient was euthyroid, as her levels of both TSH, at 1.9 uIU/mL (N 0.3–4.0), and fT4, at 16.6 pmol/L (N 10–25), were within normal limits. TPOAb, TgAb, and TRAbs tests were negative.

Further hormonal testing revealed increased testosterone at 3.52 ng/mL (N < 1), estradiol at 71.3 pg/mL (normal for her age), suppressed FSH at <0.3 mIU/mL, LH at 3.94 mIU/mL (normal for her age), and 17 OHP at 5.04 ng/mL (normal for her age).

Patient 1 presented with a tall stature and macrocephaly. The final height of Patient 1 was 172 cm (+1.0 SDS), above the mid-parental height of 160.5 cm (−0.91 SDS), with the maternal height being 164 cm (−0.3 SDS) and paternal height being 170 cm (−1.4 SDS). The patient’s head circumference was 58 cm (+2.24 SDS).

**Figure 2:**
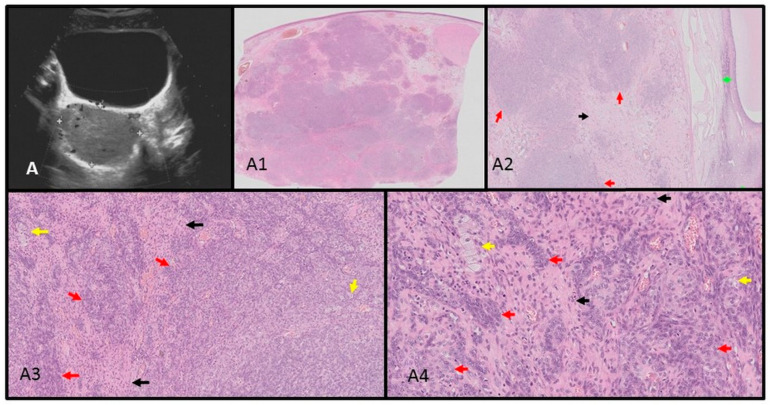
Ultrasound–histopathological assessment of SLCT moderately differentiated in Patient 1: (**A**) 5.7 × 6.9 cm solid mass in the right adnexal region seen on US scan; (**A1**–**A4**) HE histopathological assessment. The SLCT is composed of cords, solid and microcystic areas of small Sertoli cells [red arrows], as well as scattered and clustered Leydig cells with abundant eosinophilic bright-pink cytoplasm and round nuclei [yellow arrows]. Fibrous stroma [black arrows] with lymphocytic infiltration is visible. The low magnification (**A1**) shows hypo- and hypercellular nodules predominantly built by Sertoli cells presenting different architecture. The tumor was confirmed with immunohistochemistry CK AE1/AE3-positive in Sertoli cells; vimentin [+]; calretinin, CD99, and WT1-positive in part of the cells; and also EMA and CD34-negative.The proliferation index of Ki67 is approx. 40% and the mitotic figure count is 11/10 HPF [high-power field]. The tumor was totally resected with a minimal margin of 2.7 mm. In the periphery, the ovarian cortex with primary and vesicular follicles can be seen [green arrows].

Point-of-care ultrasonography was performed by the consulting endocrinologist. A thyroid ultrasound scan identified a multinodular 68 mL goiter with 15 isoechogenic cystic–solid lesions containing macrocalcifications. Vascularization was not increased in the solid parts of the nodules. An abdominal ultrasound identified a 5.7 × 6.9 cm solid mass in the right adnexal region, displacing the uterus to the left. Magnetic Resonance Imaging findings were consistent with either arrhenoblastoma or dysgerminoma. A right ovariectomy was performed, and histopathology confirmed a moderately differentiated Sertoli–Leydig cell tumor. The ultrasound–histopathological analysis is presented in Figure 2A–A4.

Hormonal tests performed 11 days after the surgery revealed an increase in estradiol to 104.1 pg/mL, disinhibition of FSH at 4.1 mIU/mL, and LH at 8.94 mIU/mL, with a normalization of testosterone to 0.30 ng/mL. Menses appeared 34 days after the surgery, with menses being regular at present.

The patient was referred to a speech therapist, whose first attempts to improve the tone of the patient’s voice were not very effective. However, six years after the surgery and consistent voice exercises, the tone of her voice is noticeably higher.

**Figure 3:**
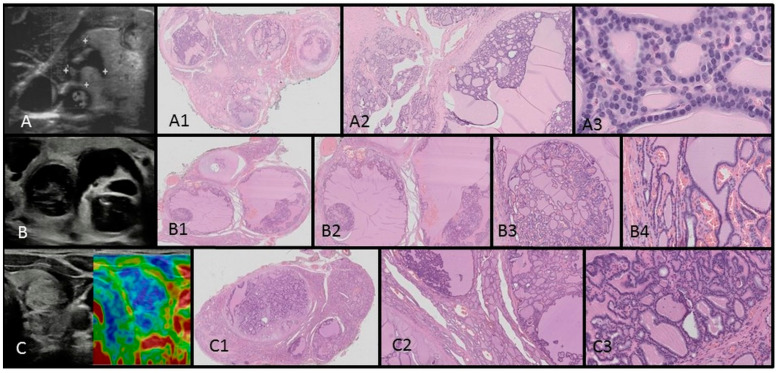
Ultrasound (US)–histopathological (HP) assessments of multinodular goiters in Patients 1 (**A**–**A3**) and 2 (**B**–**B4**,**C**–**C3**). US scans show multinodular goiters composed of isoechogenic solid–cystic nodules. In the HP assessments, the whole thyroid is composed of many hypocellular nodules filled by pink colloid. The hyperplastic nodules present a small, medium, and large vesicular structure and focal papillary arrangement. Some of the nodules show areas of non-specific granulation, fibrosis, single calcifications, and a mixed-cellular inflammatory infiltrate with foamy macrophages containing hemosiderin. The remaining thyroid parenchyma is slightly congested.

The patient was also subjected to a total thyroidectomy, for which there were two indications. Firstly, the results of FNAB fulfilled the Bethesda criteria for stage III (risk of malignancy 28%), but above all, the patient complained of dyspnea and dysphagia caused by the large tumor on her neck. Histopathology revealed benign follicular cell-derived neoplasms indicating thyroid follicular nodular disease bilaterally. The ultrasound–histopathological analysis results are presented in Figure 3A–A3. At present, the patient is euthyroid while on levothyroxine. The patient continues to undergo annual health evaluations as part of medical surveillance. Six years after the surgery, she complained of recurrent cysts of the left ovary, for which she received oral contraceptives with good results.

MNG occurrence was also detected in the patient’s mother, maternal grandmother, maternal sister, and a daughter of her maternal brother. All of these relatives including the patient’s mother were subjected to total thyroidectomies due to hoarseness. Pathological evaluations reported benign follicular cell-derived neoplasms indicating thyroid follicular nodular disease bilaterally in all cases. The presence of multinodular goiters in this family further indicated the need for genetic evaluation. Molecular analysis using NGS, confirmed with the Sanger method, revealed a pathogenic heterozygotic variant c.2953C>T [p.Gln985*] in exon 18 of the *DICER1* gene. This variant was also found in the patient’s mother, leading to a DICER1 syndrome diagnosis for both.

Unfortunately, molecular analyses were not yet performed in the other members of the patient’s family. There were no SLCTs or other components of DICER1 syndrome apart from MNGs in the patient’s family.

## 4. Case Report 2

A 12-year-old male patient was admitted to the outpatient Pediatric and Adolescent Endocrinology Department for diagnostic evaluation of a thyroid lesion. A month prior to his admission, the patient had discovered a palpable nodule in his neck, accompanied by hoarseness. An ultrasound assessment revealed structural changes throughout the thyroid gland, with multiple isoechogenic cystic–solid lesions in both lobes. His lymph nodes were unremarkable, and his levels of thyroid hormones (TSH at 1.2 uIU/mL, and fT4 at 17.4 pmol/L) and calcitonin were within normal limits. TPOAb, TgAb, and TRAbs tests were negative. Until then, the patient had been healthy, and there were no reported cases of neoplasms or thyroid issues in the family history. The patient had a tall stature of 192 cm (+2.25 SDS), above the mid-parental height of 176 cm (−0.48 SDS), and macrocephaly with a head circumference of 60 cm (+2.56 SDS).

An otorhinolaryngologist’s assessment indicated mild compression of the larynx from the left side, although vocal cord mobility was found to be normal. In the unit, biopsies from four thyroid nodules were taken, which showed atypical cells in all samples, categorized as Bethesda III. In total, ten nodules were identified, and it was decided that the patient would be placed under observation.

The patient’s perinatal history was unremarkable, with APGAR scores of 9 and 10 and a birth weight of 4130 g. The patient’s psychomotor development was normal. No genetic conditions were reported in the family’s medical history. This patient developed a multinodular goiter only. However, given the ultrasound findings, a genetic evaluation targeting *DICER1* was initiated. Molecular analysis using the NGS method, confirmed with the Sanger method, revealed a pathogenic heterozygotic variant c.2782C>T [p.Gln928*] in exon 17 of the *DICER1* gene. This variant was absent in both of the patient’s parents. Subsequently, a total thyroidectomy was performed, and the patient began thyroid hormone replacement therapy with biannual health evaluation. Histopathology revealed benign follicular cell-derived neoplasms, indicating thyroid follicular nodular disease bilaterally. Ultrasound–histopathological analysis results are presented in Figure 3B–B4,C–C3.

## 5. Discussion

### 5.1. Molecular Analyses

Molecular analysis using NGS, confirmed with the Sanger method, revealed a pathogenic heterozygotic variant c.2953C>T [p.Gln985*] in one allele of exon 18 of the *DICER1* gene [NM_177438.3 DICER1:c.2953C>T (p.Gln985*)] in Patient 1. This variant was also found in this patient’s mother. The genotype of this patient and her mother according to HGVS is ENST00000343455.7:c.[2953C>T];[=]. This variant was not found in Patient 1’s father. Other relatives of Patient 1 had not yet been molecularly assessed. A bioinformatic analysis in the Mutation Taster and Varsome programs indicated the pathogenic character of this variant. A predictive analysis on the Franklin Genoox platform (https://franklin.genoox.com, accessed on 12 March 2024), indicated that the DICER1:c.2953C>T variant was likely pathogenic: the effect on the protein being very strong (PVS1) as a null variant (stop gain) is a known mechanism of disease, and it is pathogenic moderate (PM2) in population data (extremely low frequency of this variant in gnomAD 4.0 population databases). Loss of function is also known mechanism of disease: 565 pathogenic null variants were reported in ClinVar for this gene across 26 different exons, of which 24 variants were found in exon 18. It was also confirmed with the standards and guidelines for the interpretation of sequence variants of the American College of Medical Genetics and Genomics (ACMG) and the Association for Molecular Pathology (AMP) that this variant is likely pathogenic (one PVS1 + one PM2) [27].

In Patient 2, on the basis of the clinical (ultrasound and histopathological) features of the MNG, after a literature search, a genetic evaluation targeting *DICER1* was initiated. Molecular analysis using the NGS method, confirmed with the Sanger method, revealed a pathogenic heterozygotic variant c.2782C>T [p.Gln928*] in one allele of exon 17 of the *DICER1* gene [NM_177438.3 DICER1:c.2782C>T (p.Gln928*)]. This variant has not been previously described in Ensembl but is reported once by ClinVar as a nonsense pathogenic variant, linked to a predisposition to neoplasia in DICER1 syndrome. This variant was found de novo, with the sequence not being detected in the biological mother and father, in a patient with the disease (MNG, macrocephaly, tall stature) and no family history. The genotype of Patient 2 according to HGVS is ENST00000343455.8:c.[2782C>T];[=].

A predictive analysis on the Franklin Genoox platform (https://franklin.genoox.com, accessed on 12 March 2024), indicated that the DICER1:c.2953C>T variant was pathogenic: the effect on the protein being very strong (PVS1) as a null variant (stop gain) is a known mechanism of disease, and it is pathogenic moderate (PM2) in population data (extremely low frequency of this variant in gnomAD 4.0 population databases), pathogenic moderate in de novo data (PM6), and pathogenic supporting in reputable-source data (PP5).

Loss of function is also known mechanism of disease: 565 pathogenic null variants were reported in ClinVar for this gene across 26 different exons, of which 13 variants were found in exon 17. It was also confirmed in the standards and guidelines for the interpretation of sequence variants of the ACMG and AMP in Patient 2 that this variant is pathogenic (PVS1, PM2, PM6, PP5) [27].

Therefore, it was concluded that DICER1 syndrome can be diagnosed in Patient 1 and her mother, as well as in Patient 2.

### 5.2. Ultrasound Analyses

**Figure 4:**
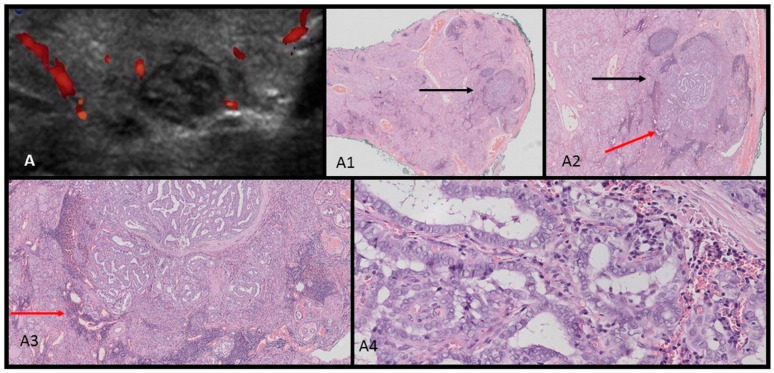
Ultrasound (US)–histopathological (HP) assessments of papillary thyroid carcinoma in an 18-year-old female patient. The US scan (**A**) shows a hypoechogenic nodule composed of multiple small nodules. In the HP images (**A1**–**A4**), a lobulated tumor [black arrow] with fibrotic layers inside invades the capsule. The outer part of the whole lesion is of a papillary–follicular structure and is surrounded by a dark-blue lymphocytic inflammation [red arrow]. The lesion is composed of polymorphic cells with nuclei showing “glassy” clearings and with grooves, invading the capsule (**A4**).

**Figure 5:**
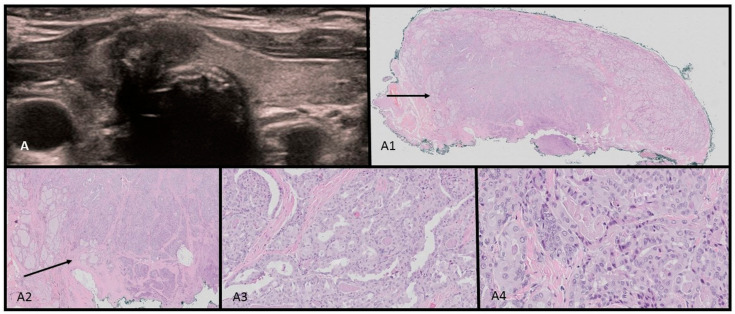
Ultrasound (US)–histopathological (HP) assessment of papillary thyroid carcinoma in a 17-year-old female patient. The US scan (**A**) shows an irregularly contoured, hypoechogenic nodule, invading the capsule. In the HE images (**A1**–**A4**), the lesion is infiltrating the thyroid parenchyma (**A1**,**A2**) and is composed of polymorphic cells with nuclei showing “glassy” clearings and with grooves (**A4**).

In this work, we present the specific ultrasound features of benign thyroid nodules in two adolescent patients that may further corroborate a DICER1 syndrome diagnosis. The typical picture of MNGs in DICER1 syndrome presented previously by Niedziela et al. is useful in ultrasonography point-of-care patient evaluation [28]. The lesions we observed were mostly cystic–solid and isoechogenic and this typical imaging picture initiated a reevaluation of other adolescent patients with MNGs from our department (unpublished data). We also observed, similar to Niedziela et al., that benign nodules have characteristic features: with multiple (≥3) mixed solid and cystic lesions, isoechogenic with macrocalcifications, and with a lack of blood flow or blood flow only in solid parts [28]. This contrasts with malignant thyroid lesions, which are usually solitary, solid, and hypoechogenic, frequently found with subcapsular localization and irregular margins, taller than they are wide with microcalcifications, and found with high intranodular blood flow [24,25,28,29,30,31]. In this study, we present these differences in Figure 4 and Figure 5, where we report a typical ultrasound picture of papillary thyroid carcinoma in two adolescent females, contrasting with the ultrasound–histopathological picture of benign lesions in DICER1 syndrome presented in Figure 3. From the clinical perspective of a pediatric endocrinologist performing ultrasonography at the point of care, these contrasting features might be useful in everyday practice. We look closely at each thyroid nodule in children, knowing that the risk of malignancy in pediatric tumors is higher than that in adults [28,29,32].

### 5.3. Histopathological Analyses

The histopathological assessments of both patients revealed that the whole thyroid was composed of multiple hypocellular nodules filled by pink colloid. The hyperplastic nodules presented a small, medium, and large vesicular structure and focal papillary arrangement. Areas of centripetal intrafollicular papillary growth (papillary adenomas) could be seen. Some of the nodules showed areas of non-specific granulation, fibrosis, single calcifications, and a mixed-cellular inflammatory infiltrate with foamy macrophages containing hemosiderin. The remaining thyroid parenchyma between the nodules was slightly congested with dilated macrofollicles. The nuclear features typical of papillary thyroid carcinomas were absent. In both patients, bilateral TFND was diagnosed.

TFND is histologically characterized by the presence of multiple bilateral nodules manifested as adenomatous- or macrofollicular-pattern nodules, well-circumscribed adenomas, and/or nodules displaying intrafollicular centripetal papillary growth (papillary hyperplasia or papillary adenomas) [33,34,35]. Barletta et al. indicated, however, that in patients presenting with adenomatous nodules, Cowden syndrome should be excluded [36]. Recently, researchers reported certain characteristic histopathological features in DICER1 syndrome [17,33,35]. In this syndrome, TFND typically exhibits a hyperplastic appearance and interestingly, Wasserman et al. noticed that the background thyroid parenchyma between the nodules showed variable involutional changes similar to alterations seen in thyrotoxicosis [17,33]. This was also confirmed in other reports [17,34]. Our patients were euthyroid (TSH at 1.9 and 1.2 uIU/mL, with normal values being 0.3–4.0), but we did not perform thyroid scintigraphy to check if the nodules were hyperfunctioning.

### 5.4. Differential Diagnosis

MNG/TFND in childhood and adolescence suggests possible CPS and as presented in recent reports, a pathogenic *DICER1* variant is the causative factor in a majority of cases involving young patients [11,18,20]. However, differential diagnosis must also exclude other syndromes, such as intestinal polyposis syndromes, Cowden/PTEN hamartoma tumor syndrome, Carney complex, Werner’s syndrome, Pendred syndrome, McCune–Albright syndrome, and familial medullary and papillary thyroid carcinomas, among others [1,11,20,21].

### 5.5. Surveillance Recommendations

Schultz at al. and Schneider et al. published surveillance recommendations for carriers of pathogenic DICER1 variants [22,23].

Schultz et al. recommend considering germline *DICER1* genetic testing in an individual with a personal history of at least one major or two minor indications for testing, in children < 7 years of age, who are at the greatest risk of PPB, and in second- and third-degree relatives, especially if they are parents of young children [23].

According to the surveillance strategies recommended by Schultz et al., both of our patients fulfilled the major criteria for DICER1 molecular testing [23]. In Patient 1, we found three major indications: ovarian SLCT, MNG, and a positive family history, as well as childhood-onset MNG. In Patient 2, there was one major (childhood-onset MNG) and two minor (macrocephaly, MNG) criteria fulfilled.

### 5.6. Limitations

There are several limitations to our report. We currently present only two patients with *DICER1* pathogenic variants. We were unable to perform functional testing; however, ACMG classification reports that both variants are null variants, having a strong negative effect on the protein.

In Patient 1, this variant segregates with disease present on the maternal site of the family. Unfortunately, we could not molecularly evaluate all of the members of her family, apart from Patient 1’s mother and father. Also, we could not perform sequencing of the ovarian tumor and thyroid tissues due to unavailable resources to check for somatic *DICER1* variants.

Our approach to both patients included a thyroidectomy due to hoarseness. Other reports recommend that ultrasound surveillance could be chosen in patients with benign cytology [20,28]. Surveillance is important, as Rutter et al. reported a family with a germline *DICER1* pathogenic variant whose members developed small foci of DTC within MNGs, and these patients were not exposed to chemotherapy or radiotherapy previously [37].

### 5.7. Strengths

The strengths of this report include our comprehensive clinical approach to these patients from detecting lesions in the thyroid and ovary, the presentation of unique solid–cystic lesions in the thyroid, the detection of typical histological features of nodules that raised our concern about possible CPS, and the presentation of hormonal changes before and after surgery and further patient follow-ups.

In presenting the first patient, we would like to highlight the symptoms of androgen excess in adolescent patients such as acne, hirsutism, irregular menses, balding, clitoromegaly, decreased breast tissue, and deepening of the voice. Differential diagnosis should exclude non-classic congenital adrenal hyperplasia, the most common etiology, and should subsequently evaluate possible adrenal or gonadal sources of androgens.

SLCTs are classified into three subtypes: the *DICER1* variant (younger patient age), *FOXL2* variant, and *DICER1*/*FOXL2* wildtype variant [38]. SLCTs, due to *DICER1* alterations, are probably more common in patients with androgenic symptoms [39]. From the point of view of a pediatric endocrinologist, it is interesting to understand the mechanism of the formation of Sertoli and Leydig cells in the ovary. Research groups have demonstrated that *DICER1* alterations may downregulate ovarian development genes in ovarian pregranulosa cells, such as FST and CYP19A1, and upregulate Sertoli cell differentiation genes, FGF9 and FGFR2, known to sustain the expression of SOX9 [3,40,41].

## 6. Conclusions

Recent advances in genetic evaluation and in histological approaches indicate that MNG/TFND, although rare in the pediatric population, when accompanied by characteristic ultrasound and histopathological features, and by additional features such as androgenization, may warrant additional assessment of the *DICER1* gene within CPS molecular-panel screening.

## Data Availability

The data presented in this study are available on request from the corresponding author. The data are not publicly available to ensure the confidentiality of the patients’ personal information.
